# Online Interpersonal Sexual Objectification Experiences and Teenage Girls’ Self-Objectification: The Role of Broad Conceptualization of Beauty

**DOI:** 10.3390/bs12070210

**Published:** 2022-06-24

**Authors:** Sen Lin, Liming Li, Libiao Jiang

**Affiliations:** 1 School of Humanities and Social Sciences, Xi’an Jiaotong University, Xi’an 710049, China; liliming@mail.xjtu.edu.cn (L.L.); jlb8582@126.com (L.J.); 2 Department of Physical Education, Shaanxi University of Science & Technology, Xi’an 710049, China

**Keywords:** online interpersonal sexual objectification, self-objectification, broad conceptualization of beauty, teenage girls, moderating effect

## Abstract

Self-objectification is a common and deleterious phenomenon among young teenage girls, for which interpersonal sexual objectification experiences are a great risk; in the current information era, sexual objectification experiences may also expand into the online space. Based on this, this study aimed to examine the association between online interpersonal sexual objectification (OISO) experiences and teenage girls’ self-objectification, as well as the potential moderating role of broad conceptualization of beauty in relation to this. Seven hundred and seventy-one female undergraduate students were recruited voluntarily to complete questionnaires on OISO experiences, self-objectification, and the broad conceptualization of beauty. Results indicated that OISO experiences were positively associated with teenage girls; self-objectification and the broad conceptualization of beauty could significantly buffer this relation, which was weakened among individuals with a high level of broad conceptualization of beauty. This study expands previous research on sexual objectification, providing practical significance for promoting the well-being of teenage girls.

## 1. Introduction

Physical appearance is an important contributor to individual self-conception, especially among females [1]. However, excessive focus on physical appearance (such as self-objectification) could be harmful [2,3]. Self-objectification refers to the phenomenon that individuals (mainly females) focus on their physical appearance rather than on what they can do or how they feel, which is usually operationalized as the degree to which women’s self-descriptions emphasize their physical attractiveness [4]. It is not only a common phenomenon but also a great risk to female health and well-being worldwide. Studies conducted in different countries all found that self-objectification was closely associated with physical and mental health problems, such as eating disorders, sexual dysfunction, substance abuse, body dissatisfaction, and consequent anxiety and depression [5,6,7,8,9].

Against this background, the factors affecting self-objectification have been widely examined. According to objectification theory, sexual objectification experiences are the direct and important risk factors for females’ self-objectification, among which sexually objectifying media content (especially the presentation of thin or sexy images presented in the media) and interpersonal experiences (e.g., teasing, comments, or evaluations on appearance and body parts, and being looked or stared at in interpersonal interaction) are the most prominent factors [8,10,11]. Interpersonal sexual objectification experiences not only increased the risk of self-objectification but were also closely associated with various adverse outcomes (e.g., body dissatisfaction and restrictive eating behaviors) [12,13]. Furthermore, empirical studies also indicated that females were more likely to encounter sexual objectification experiences in daily life—the sexy images (mainly reflecting the beautiful/attractive appearance features or body shape) presented in the media were mainly females, and the sexual objectification experiences females encountered were nearly four times as many as those of males [14,15].

In the current information era, the Internet and various applications (social networking sites and instant messaging, for example) have become popular platforms for interpersonal interaction and information access [16,17]. Thus, sexual objectification experiences may also expand into the online space. Relevant studies mainly focused on limited online objectification experiences (objectifying media content online). They found that photo-related activities, such as body image comparisons online and viewing and posting selfies, may boost self-objectification [6,18,19]. However, online interpersonal objectification experiences (OISO experiences, referring to the experience of being subjected to sexual objectification in online interpersonal communication) require further examination, especially those OISO experiences that are ubiquitous online [12]. For example, females often perceive the requirement to post or send selfies or photos because others are highly interested in how they look, and they frequently encounter appearance-related feedback [20,21]. Thus, based on these discussions, this study aimed to examine females’ OISO experiences and their relation to self-objectification, and it was hypothesized that OISO experiences were positively associated with self-objectification (H1).

In addition, the moderating mechanism underlying this relation is of great theoretical and practical significance, which should be examined. In particular, the organism–environment interaction model indicates that individuals are not equally influenced by the same environmental contexts and that individuals with certain personality traits would be less influenced [22]. Regarding body image, positive notions and attitudes toward body image (such as body appreciation, body image flexibility, and broad conceptualization of beauty) have received more attention in recent years [23,24,25], and the cognitive-behavioral process model of body image [26] also stresses the key role of cognitive mechanisms in the connection between exposure to body image-related threats or challenges (distal factors) and body perception [27]. Empirical studies determined that positive notions and attitudes about body image (such as body appreciation and body image flexibility) could buffer the influences of sexual objectification experiences on individuals’ body image concerns [2,28].

The broad conceptualization of beauty refers to a flexible and broad-ranging definition of beauty, with an appreciation for different appearances and styles, indicating that beauty is reflected in terms of both internal and external characteristics; it was found to be closely associated with other positive notions and attitudes on body image outcomes (e.g., body satisfaction and acceptance, body appreciation, and body image flexibility) [29,30]. Thus, it would make individuals attach less importance to body image (namely, focusing on such inner positivity as self-confidence and intelligence rather than external characteristics) and further resist the impact of sociocultural ideals and standards. Studies also found it was positively correlated with adaptation, such as body satisfaction, self-esteem, and life satisfaction, while negatively correlated with thin-ideal internalization (referring to the extent to which individuals subscribe to social standards for physical appearance and aspire to attain these standards), body comparisons, contemplation of cosmetic surgery, and self-objectification [11,30,31]. Based on this evidence, it was hypothesized that a broad conceptualization of beauty could buffer the relationship between ISO experiences and self-objectification (H2).

Therefore, based on the objectification theory, the background of the current information era, and relevant studies, a moderating model was constructed to examine the association between online interpersonal sexual objectification (OISO) experiences and teenage girls’ self-objectification, and its underlying moderating mechanism—whether a broad conceptualization of beauty could moderate this relationship.

## 2. Materials and Methods

### 2.1. Participants and Procedure

A convenience sampling method was adopted to recruit participants from the students attending the courses on psychological health education at a public university. Through this procedure, a sample of 827 female undergraduate students was recruited to complete a paper-pencil questionnaire (which could be completed in five minutes) after signing the informed consent. Finally, 771 participants (all heterosexual) completed the survey, with an age range from 18 to 23 years old (Mage = 19.78 ± 1.88); 56 female students dropped out as more than 30% of the items were not answered or involved repeated, regular values.

### 2.2. Measurement

*Online interpersonal sexual objectification (OISO).* The online interpersonal sexual objectification scale developed by Luo et al. [12] was adopted. It was a unidimensional scale designed to measure participants’ OISO experiences using six items (e.g., “How often have you noticed someone evaluating your size or appearance in your photo album on social networking sites such as Q-zone and WeChat Moments?”). Participants were asked to respond on a 5-point scale ranging from 1 “Never” to 5 “Always”, with higher scores indicating more OISO experiences. In this study, the confirmatory factor analysis revealed an acceptable fit: *χ*^2^/*df* = 4.06, RMSEA = 0.06, NFI = 0.96, CFI = 0.98, the Cronbach’s α was 0.83.

*Broad conceptualization of beauty.* The broad conceptualization of the beauty scale [31] developed by Tylka and Iannantuono with nine items (e.g., “A woman’s confidence level can change my perception of her physical beauty”) was adopted, which has been translated and used in Chinese samples with good validity and reliability. Participants were asked to respond to each item with a 5-point scale ranging from 1 “Strongly Disagree” to 5 “Strongly Agree”, with higher scores indicating a more flexible definition of beauty. In this study, the confirmatory factor analysis revealed an acceptable fit: *χ*^2^/*df* = 5.22, RMSEA = 0.07, NFI = 0.94, CFI = 0.95, the Cronbach’s α was 0.88.

*Self-objectification.* The objectified body consciousness scale was adopted to measure self-objectification. It mainly focuses on the experiences, feelings, and beliefs closely associated with the notion that their body is an object and contains 24 items to measure three dimensions—body shame, body surveillance, and appearance control beliefs. It has been widely used in relevant research and has also been found to have good validity and reliability among young Chinese women [32]. Participants were asked to respond on a 7-point scale ranging from 1 “Completely Disagree” to 7 “Completely Agree”, with a higher average total score indicating a higher level of self-objectification. In this study, the confirmatory factor analysis revealed an acceptable fit: *χ*^2^/*df* = 4.85, RMSEA = 0.07, NFI = 0.96, CFI = 0.97; the Cronbach’s α for the three dimensions (body shame, body surveillance, and appearance control beliefs) and the whole scale were 0.89, 0.86, 0.84, and 0.91, respectively.

### 2.3. Ethics Approval

This study was approved by the Ethical Committee for Scientific Research at the researchers’ affiliated institution. The ethical values required in research with human beings, the fundamental principles included in the Helsinki Declaration (e.g., informed consent, protection of personal data, and guarantees of confidentiality), as well as the regulations of the education management department were followed. At the same time, all of the participants who took part in the study were told about the goals of the study, and they also gave oral consent.

### 2.4. Statistical Analysis

All the data were analyzed using SPSS 23.0. First, the common method bias was tested. Then, the descriptive statistics and correlational analyses were calculated. Lastly, the PROCESS macro (http://www.afhayes.com, accessed on 15 August 2021) for SPSS (Model 1), suggested by Hayes [33], was adopted to test the moderating model with 5000 bias-corrected samples (the moderating effect is significant when 95% CI does not include zero). In addition, referring to relevant studies [6,12], age, body mass index (BMI), and the general online communication use (participants reported the time spent on a regular day with hours as an indicator) were included as control variables to exclude their potential influences on the associations among main variables.

## 3. Results

### 3.1. Test for Common Method Bias and Normality

According to relevant suggestions, because all of the study data was collected via self-reported questionnaires, there could be common method bias. Thus, confirmatory factor analysis was conducted to test the common method bias [34]. The result showed a poor model fit: *χ*^2^/*df* = 19.56, RMSEA = 0.22, TLI = 0.51, CFI = 0.55), indicating that the current study had no significant issue with common method biases.

Then, the Kolmogorov–Smirnov test was adopted to check the normality of the research data. In the current study, all the skewness values were below 2.0, and all the kurtosis values were below 7.0, which indicated that the data was normally distributed.

### 3.2. Descriptive Statistics and Correlations between Main Study Variables

Table 1 presents the means, standard deviations, and Pearson’s correlations among the main variables. As indicated, all the main research variables were significantly correlated with each other—OISO experiences were positively correlated with self-objectification (*r* = 0.32), while both of them were negatively correlated with the broad conceptualization of beauty (*r* = −0.15; *r* = −0.47). This suggested that it was suitable to further test the potential moderating effect of the broad conceptualization of beauty.

### 3.3. Testing the Hypothesized Moderating Model

**Figure 1:**
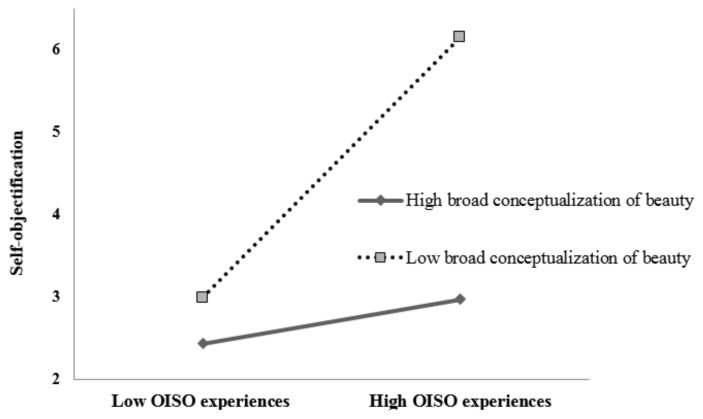
Moderating effect of broad conceptualization of beauty in the relationship between OISO experiences and self-objectification.

The PROCESS macro for SPSS (Model 1) was used to test the proposed hypotheses, with age, BMI, and general online communication use included as control variables. OISO experiences, broad conceptualization of beauty, and the interaction of them all were entered into the regression model, and the results found that OISO experiences (*β* = 0.13, *p* < 0.001, 95% CI = 0.04; 0.20) showed a significant positive association with self-objectification, while broad conceptualization of beauty (*β* = −0.19, *p* < 0.001, 95% CI = −0.30; −0.10) showed a significantly negative association with self-objectification; in particular, the interaction of OISO experiences and broad conceptualization of beauty was also significantly associated with self-objectification (*β* = −0.14, *p* < 0.001, 95% CI = −0.22; −0.06), indicating that the moderating effect of a broad conceptualization of beauty was significant. Then, conditional direct effects (based on the moderator values at 1SD below and above the mean) further examined the moderating effect (see Figure 1), which showed that the association between OISO experiences and self-objectification was more significant among students with a low level of broad conceptualization of beauty (1 SD below the mean; *β* = 0.31, *p* < 0.001, 95% CI = 0.20; 0.45) than among students with a high level of broad conceptualization of beauty (1 SD above the mean; *β* = 0.09, *p* < 0.05, 95% CI = 0.02; 0.15). Thus, individuals having OISO experiences are at higher risk of self-objectification, and a broad conceptualization of beauty could buffer the influence of OISO experiences on self-objectification.

## 4. Discussion

From the perspective of objectification theory and the background of the current information era, based on relevant evidence, this study sought to examine the association between OISO experiences and teenage girls’ self-objectification, as well as its underlying mechanism—the buffering effect of a broad conceptualization of beauty in this relation.

Firstly, consistent with the main points of objectification theory [8,11], the results indicated a positive association between the OISO experiences and teenage girls’ self-objectification. Based on related studies revealing the influences of sexual objectification and interpersonal experiences of self-objectification in real life [16,18], this finding may further reveal that interpersonal objectification experiences also exist in the online space and encountering more OISO experiences may further increase the risk of individuals’ self-objectification. Though differences exist between the interpersonal objectification experiences online and in real life, both of them would make individuals attach more importance to their body appearance [6,12]. Thus, similar to the experiences in real life, the OISO experiences (e.g., asking to send selfies or photos and appearance comments in online interpersonal interaction) are also risk factors increasing individuals’ sexual objectification. Especially due to the features of online communication, such as anonymity and disinhibition, girls are more likely to encounter interpersonal objectification experiences when using the Internet [20,35]. At the same time, previous studies have examined the photo-related activities in online communication (e.g., posting selfies and viewing others’ photos) and found these activities and experiences were closely associated with body dissatisfaction and concerns, as well as eating disorders [2,19,36], which are closely associated with self-objectification. This study may integrate and expand these findings by focusing on sexual objectification experiences online.

Secondly, this study further examined the underlying moderating mechanism, specifically the buffering role of a broad conceptualization of beauty, and the results found that the association between OISO experiences and self-objectification was weakened among students with a high level of broad conceptualization of beauty. The cognitive-behavioral process model of body image suggested that cognitive factors are the key to determining individuals’ body image-related adaptation when facing outer threats [26,27]. Empirically, positive notions and attitudes toward body image, such as body appreciation and body image flexibility, have been found to be not only positively associated with healthy body perception and eating behaviors [30,31] but also could act as protection against the deleterious influences of objectification experiences [2,28]. Regarding the broad conceptualization of beauty, it enables individuals to hold a positive and flexible attitude toward body image and beauty [23,24,30], making them view body image with an open mind, and perceive a wider range of body appearances as beautiful; at the same time, individuals with a higher level of broad conceptualization of beauty tend to focus on inner positivity (e.g., self-confidence, and intelligence) rather than external characteristics (e.g., appearance attractiveness). Thus, individuals endorsing a broad conceptualization of beauty would process body-related information positively, and they are less likely to engage in appearance-related activities (such as body comparison) and be affected by the societal appearance ideals. Empirical studies also found that broad conceptualization of beauty was positively correlated with positive body image outcomes (e.g., body satisfaction and acceptance, body appreciation, and body image flexibility), while negatively associated with the body and eating-related concerns [11,29,31]. Based on this evidence, female students with a high level of broad conceptualization of beauty would pay less attention to appearance and be less likely to engage in body-related activities and appearance online (including interpersonal objectification experiences), while being less affected by the OISO experiences. Namely, a broad conceptualization of beauty could alleviate the deleterious influence of OISO experiences on self-objectification.

### Limitations of the Study

Firstly, similarities and differences between the sexual objectification experiences online and in real life may exist, so their uniqueness and the interaction between them should be further examined. Secondly, due to the limitations of the cross-sectional design and cultural differences in the beauty and social pressure on body image [37], causal inference designs should be adopted and participants from multicultural backgrounds should be studied to provide additional insights. Thirdly, other objectification experiences and underlying mechanisms should be considered to examine this theme more clearly and comprehensively; at the same time, intervention studies are of great need in the future. 

## 5. Conclusions

To sum up, this study found that OISO experiences were positively associated with teenage girls’ self-objectification and that a broad conceptualization of beauty could significantly buffer this relation—this relation was weakened among individuals with a high level of broad conceptualization of beauty. This study has several theoretical and practical implications. Theoretically, this study firstly expanded on previous studies on self-objectification by examining interpersonal sexual objectification experiences online, and found that interpersonal objectification experiences also exist in the online space, and having OISO experiences may boost the risk of individuals’ self-objectification. This could also deepen our understanding of sexual objectification experiences, the influences of online communication, and the risk factors of self-objectification. Secondly, the results also expanded the objectification theory, suggesting that it is suitable to explain the relevant issues in the online space. Lastly, this study also contributes to revealing the underlying mechanism of self-objectification and body-related concerns. Practically, the results could facilitate ideas for healthy Internet use and the intervention of self-objectification and related issues. Firstly, young teenage girls should acknowledge the potential deleterious influences of OISO experiences; more importantly, they also should know how to deal with these experiences, for example, rejecting messages containing objectifying content or individuals who emphasize or evaluate their physical appearance. Then, considering the moderating role of the broad conceptualization of beauty, relevant interventions should focus on positive notions such as broad conceptualization of beauty and body appreciation, and programs should be developed and adopted to enhance these positive notions, so as to alleviate or block the influences of various sexual objectification experiences and further decrease the risk of self-objectification and its negative outcomes (including body image disturbance and eating disorder symptoms).

## Figures and Tables

**Table 1:** Descriptive statistics and correlations among study variables.

Variable	*M* (*SD*)	1	2	3	4	5	6
1. Age	19.78 (1.88)	1					
2. General online communication use	1.45 (0.95)	0.12 **	1				
3. BMI	18.69 (2.02)	−0.05	−0.03	1			
4. OISO experiences	2.28 (0.86)	−0.09 *	0.18 ***	−0.08 *	1		
5. BCOB	3.44 (0.88)	−0.05	−0.08 *	0.06	−0.15 ***	1	
6. Self-objectification	4.14 (0.63)	0.10 **	0.14 **	0.02	0.32 ***	−0.47 ***	1

Note: BMI = Body mass index; OISO experiences = Online interpersonal sexual objectification experiences; BCOB = Broad conceptualization of beauty; * *p* < 0.05, ** *p* < 0.01, *** *p* < 0.001.

## Data Availability

The data of this study are available from the corresponding author upon reasonable request.
